# The Mediation Effect of Nurses’ Artificial Intelligence Literacy Between Professional Self‐Concept and Evidence‐Based Practice: A Cross‐Sectional Study

**DOI:** 10.1155/jonm/3840628

**Published:** 2026-05-28

**Authors:** Shanwei Li, Liping Wu, Yan Tang

**Affiliations:** ^1^ Department of General Surgery and Trauma Surgery, Children’s Hospital of Chongqing Medical University, National Clinical Research Center for Child Health and Disorders, Chongqing Key Laboratory of Structural Birth Defect and Reconstruction, Ministry of Education Key Laboratory of Child Development and Disorders, Chongqing, 400014, China, moe.edu.cn; ^2^ Department of Nursing, Children’s Hospital of Chongqing Medical University, Chongqing, 400014, China, chcmu.com

**Keywords:** artificial intelligence literacy, evidence-based practice, mediation effect, nursing education, professional self-concept

## Abstract

**Background:**

AI technology has had a significant revolutionary impact on the fields of healthcare and education. For the nursing staff population, the lack of artificial intelligence (AI) literacy may not only weaken the construction of their professional self‐concept but also constrain the development of evidence‐based practice. However, empirical research on the intrinsic correlation mechanism between these three factors is still relatively scarce at present.

**Aim:**

The purpose of this study was to explore the mediating role of AI literacy in the relationship between nurses’ professional self‐concept and evidence‐based practice.

**Methods:**

A cross‐sectional study was conducted from October 15 to November 1, 2025, using convenience sampling to select 497 nurses from four tertiary public hospitals in Chongqing. The data collection tools include participant demographic characteristics, AI Literacy Scale (AILS), Nurse Self‐Concept Questionnaire (NSCQ), and Evidence‐Based Practice Questionnaire (EBPQ). The statistical software *R* (version 4.5.2) was adopted for data analysis, which included data feature description, correlation verification, and structural equation modeling.

**Results:**

The overall demographic characteristics of the respondents were characterized by high educational levels, a mix of middle‐aged and young people, and extensive work experience. The average scores for professional self‐concept, AI literacy, and evidence‐based practice were 232.29 ± 42.57, 63.34 ± 10.14, and 143.05 ± 22.45, respectively. It was found that a positive relationship exists between nurse AI literacy and professional self‐concept (*r* = 0.89, *p* < 0.001), as well as between nurse professional self‐concept and evidence‐based practice (*r* = 0.94, *p* < 0.001). A significant positive correlation has also been found between AI literacy and evidence‐based practice (*r* = 0.92, *p* < 0.001). AI literacy played a partial mediating role between nurse professional self‐concept and evidence‐based practice, with a mediation effect value of 0.587 (95% CI: 0.569–0.606), which explained 38.5% of the total effect.

**Conclusion:**

The study confirmed that there was a positive relationship between nurse professional self‐concept and evidence‐based practice, and AI literacy played a partial mediating effect in this relationship chain. It can be seen that AI literacy plays an indispensable and critical role in promoting the shape of nurse professional self‐concept and enhancing their evidence‐based practice ability.

**Implications for Nursing Management:**

Improving the AI literacy of nurses and conducting precise training are fundamental tasks in promoting the effective empowerment of clinical nursing scenarios with AI technology. To this end, it is necessary to integrate knowledge and skills related to AI into the nursing education system and simultaneously promote the lifelong professional development of nurses to effectively enhance their ability to use AI technology to optimize medical services. At the same time, healthcare institutions and nursing managers should focus on building supportive practice environments, advocating for standardized clinical applications of AI technology, and always adhering to the nursing core values guided by patient needs.

**Trial Registration:** Chinese Clinical Trial Registry: ChiCTR2600118905

## 1. Introduction

The accelerated advancement of artificial intelligence (AI) is fundamentally reshaping the global clinical practice paradigm, and its core values can be condensed into three dimensions: accurately improving disease diagnosis efficiency, reducing misdiagnosis, and missed diagnosis risks through intelligent analysis methods; continuously optimizing the quality of patient care, relying on technology to empower and improve the service experience of the entire diagnosis and treatment process; scientific support for clinical decision‐making provides objective basis for the selection of individualized treatment plans [1, 2]. Against the backdrop of AI gradually integrating into the medical service system and becoming a core supporting element, the work mode and capability requirements in the nursing field naturally need to be innovated accordingly. This change not only requires nursing staff to master the practical application methods of AI‐related technologies but also to establish confidence and awareness in actively using intelligent tools. Only in this way can we ensure that the technology is truly implemented in clinical nursing scenarios and plays an effective role [3, 4].

It is worth noting that in the medical environment deeply empowered by AI, nursing staff need to focus on cultivating two core abilities, which together form the cornerstone of high‐quality nursing services: one is professional self‐concept, which is manifested in the clear understanding and high recognition of nursing staff’s professional abilities, as well as their subjective initiative to actively explore innovative solutions when facing clinical nursing difficulties; the second is evidence‐based practice, which can help nursing staff overcome the limitations of empiricism and make scientific and reasonable nursing decisions in complex and uncertain clinical situations [5, 6]. The cultivation of these two core competencies is directly related to the AI literacy of nursing staff. Good AI literacy can fully stimulate the enthusiasm of nursing staff to learn new technologies and apply intelligent tools, enhance their willingness to accept technological innovations in the medical field, and thus build a firm groundwork for systematic improvement of clinical nursing quality and the professional development of nursing staff themselves [7, 8].

AI literacy refers to the comprehensive competence of an individual to understand the principles of AI technologies, apply relevant products in practice, and evaluate their practical utility, thereby facilitating the efficient execution of various professional and daily tasks [9, 10]. As the main beneficiaries of prospective AI applications and the core force underpinning future healthcare delivery, the advancement of AI literacy among nurses has a dual significance: on the one hand, it plays a pivotal role in spearheading and facilitating the implementation and advancement of AI within the nursing domain; in addition, it is directly related to the career development prospects of individual nurses [11, 12]. Research has shown that improving AI literacy can enhance nurses’ professional self‐awareness. Nurses with strong AI application capabilities are often able to efficiently complete multiple tasks: they can not only analyze complex clinical data but also collaborate closely with interdisciplinary teams to explore innovative solutions to practical nursing‐related challenges and demonstrate their occupational expertise and career growth prospects [13, 14].

Professional self‐concept is the core cognitive and emotional experience of nurses toward their professional identity, which is deeply influenced by the unique knowledge system, professional norms, and values of the nursing discipline [15, 16]. This concept not only shapes nurses’ professional thinking patterns, behavioral norms, and emotional response styles but also becomes an important psychological resource for them to cope with technological changes. In the context of the rapid iteration of medical technology, professional self‐concept holds dual value for the adaptive development of nurses. On the one hand, it helps nurses effectively integrate emerging technologies such as AI and transform them into tools to improve nursing quality; furthermore, it encourages nurses to be guided by innovative thinking and humanistic care, better meeting the increasingly diverse health needs of patients [17, 18]. Available studies have confirmed that nursing staff with a more robust professional self‐concept tend to display greater proactivity in clinical practice. They are more actively involved in multidisciplinary collaboration for complex cases, actively applying evidence‐based support provided by AI, and continuously exploring optimization solutions for nursing processes. It is worth noting that improving nurses’ AI literacy can form a virtuous cycle by enhancing the confidence in using intelligent technology to solve clinical problems, thereby strengthening their professional self‐identity and ultimately promoting the development of nursing practice toward a more efficient and humane direction [19, 20].

Evidence‐based practice is a comprehensive ability developed by nurses as they integrate diverse professional knowledge, practical skills, and professional ethics. It is specifically reflected in the process of providing nursing services to specific patients, groups, or communities, accurately extracting practical problems with clinical value, and conducting relevant information retrieval, screening, evaluation, and transformation applications through multiple channels [21, 22]. Its core goal is to help nurses proficiently master the professional skills required for the entire process of evidence‐based practice. AI technology can leverage the real‐time collection of patient clinical data, intelligent risk assessment frameworks, and patient‐care decision‐aiding tools to provide scientific and efficient technical support for nurses’ evidence‐based practice, thereby enabling nursing staff to make clinical judgments more quickly and accurately [23, 24].

Although existing studies have explored the associations between AI literacy, professional self‐concept, and evidence‐based practice among nurses separately [25, 26], the literature gap remains clear: few empirical studies have systematically examined the three‐way interaction mechanism, especially the mediating role of AI literacy in the pathway from professional self‐concept to evidence‐based practice. Most research focuses on single‐factor effects or dual correlations, lacking an integrated structural model to explain how AI literacy links nurses’ professional identity cognition with their evidence‐based clinical behavior. Therefore, the purpose of this study was to explore the mediating role of AI literacy in the relationship between nurses’ professional self‐concept and evidence‐based practice. Against the background of rapid integration of AI into smart healthcare, this study fills the theoretical gap in the collaborative research framework of “AI literacy–professional cognition–practical ability.” It provides an empirically validated framework for nursing education and management and offers evidence‐based guidance for cultivating AI‐proficient, self‐confident, and practice‐oriented nurses to adapt to the development of intelligent medical care.

## 2. Methods

### 2.1. Study Design

A cross‐sectional survey method was adopted in this study to explore the interrelationship between AI literacy, professional self‐concept, and evidence‐based practice based on a predetermined research framework. It strictly follows the relevant standards of the Strengthening the Reporting of Observational Studies in Epidemiology (STROBE) to ensure the standardization of the research implementation process and accurate recording of results [27, 28].

### 2.2. Participants and Sample

With convenience sampling adopted as the sampling method, this study launched a cross‐sectional survey among frontline nursing practitioners in four tertiary public hospitals in Chongqing from October 15 to November 1, 2025. The inclusion criteria are (1) registered nursing staffs in possession of valid nursing practicing certificates; (2) dedicated to clinical nursing work; and (3) engage in this study voluntarily and execute a written informed consent document. The exclusion criteria are (1) informally employed nurses, practicing nurses from other hospitals, and intern nursing students; (2) nursing staff not scheduled for work throughout the investigation phase; and (3) nurses with physical and mental health issues that require treatment. As a methodological limitation, convenience sampling may reduce sample representativeness and restrict the generalizability of the study findings.

G∗Power 3.1 software was employed to perform the calculation of sample size, which is specifically designed for multiple linear regression analysis. In the course of calculation, an effect size of 0.15 was specified, a significance level of 0.05 was fixed, and a test power of 0.90 was defined, with the influence of 14 independent variables taken into full consideration [29, 30]. At the same time, the preset invalid questionnaire ratio was 20% due to incomplete surveys or insufficient filling time. Based on the above parameters, the computation results indicated that the necessary sample size stood at 385. In the actual survey, 497 questionnaires were retrieved altogether. After excluding invalid responses with logical contradictions, duplicate answers, or abnormal filling time, 26 invalid questionnaires were removed, resulting in a final valid sample size of 471, corresponding to a valid response rate of 95%, which met the predetermined sample size requirement.

### 2.3. Measures

1. General characteristic questionnaire. Formulated independently by the investigators, the questionnaire was composed of 12 items, including sex, age, work tenure, educational background, department, professional rank, job position, clinical teacher status, marital status, number of children, graveyard shift, and training experience at or above the college level in the past 3 years.
2. AI Literacy Scale (AILS). The AILS is a Chinese version scale developed by Wang et al. [31], designed to assess AI literacy in the general population. This scale covers four distinct dimensions, namely, evaluation, awareness, ethics, and usage. Every dimension has three measurement items, and the scale consists of 12 items altogether. The scale adopts a 7‐point Likert scoring method, with a scoring range of 1–7 points, corresponding to attitudes ranging from “strongly disagree” to “strongly agree.” The overall score of the scale covers the interval of 12–84 points, where elevated scores reflect better AI literacy among the subjects. Cronbach’s *α* coefficient corresponding to the target scale in this study was measured at 0.810.
3. Nurse Self‐Concept Questionnaire (NSCQ). The NSCQ was developed by Cowin [32] specifically for evaluating the professional self‐concept level of clinical nurses. For the present study, we used the Chinese revised version, which has been Sinicized and validated for validity [33]. This scale includes six dimensions: leadership, communications, care, self‐esteem, and communication with colleagues. Each dimension has 8, 6, 5, 6, 5, and 6 measurement items, respectively, which sum up to 36 items in total. The scale adopts an 8‐point Likert scoring method, with a scoring range of 1–8 points, corresponding to attitudes ranging from “very wrong” to “very correct”; the overall score of the scale covers the interval of 36–288 points, where greater scores signify a more prominent professional self‐concept level among nursing staff. Cronbach’s α coefficient corresponding to the target scale in this study was measured at 0.985.
4. Evidence‐Based Practice Questionnaire (EBPQ). The EBPQ was developed and compiled by Ruzafa‐Martínez et al. [34]. Its core purpose is to assess the level of evidence‐based practice among clinical nursing practitioners. This study adopted a Chinese‐language version that has been localized and validated for reliability and validity [35]. This scale covers four dimensions: attitude, knowledge, skills, and utilization, with 10, 10, 9, and 6 measurement items set for each dimension, totaling 35 items. The questionnaire adopts a 5‐point Likert scoring method, where the rating interval covers 1–5 points, matching the attitudinal spectrum from “completely disagree” to “completely agree”; the aggregate score of the scale ranges between 35 and 175 points, where greater scores signify stronger evidence‐based practice capability in clinical nursing practitioners. Cronbach’s *α* coefficient corresponding to the target scale in this study was measured at 0.970.

### 2.4. Data Collection

Before the investigation is officially launched, the researchers first uploaded the designed survey questionnaire to the online platform *Questionnaire Star* and generated the corresponding QR code. Subsequently, they submitted the QR code and questionnaire explanation materials to nursing department managers across all participating hospitals, detailing the survey objectives, implementation process, and precautions to obtain their support and cooperation. After obtaining consent, the head of the nursing department forwarded the QR code to the internal nurse community of the hospital for participation in the investigation. To ensure the standardization and effectiveness of data collection, all questionnaires were filled out anonymously, and instructions were uniformly attached at the beginning of the questionnaire, encompassing the research objectives, practical significance, and key considerations for accurate completion. At the same time, all questionnaire items were set as mandatory fields to avoid missing values, ensure data integrity, and maintain the reliability of subsequent statistical analysis, and the same IP address can only submit the questionnaire once through technical means. After the survey was completed, the researchers exported all completed questionnaire data from the platform backend for systematic review and screening. The specific operation includes removing invalid questionnaires with logical contradictions, duplicate answers, or abnormal filling time, and ultimately retaining valid data for subsequent analysis.

### 2.5. Ethical Considerations

This study was approved by the Ethics Committee of the Children’s Hospital of Chongqing Medical University (approval number: 368/2025). The entire research process strictly adheres to the ethical guidelines of the Helsinki Declaration. As a cross‐sectional observational study, it was registered in the Chinese Clinical Trial Registry (ChiCTR2600118905) to ensure protocol transparency, standardized implementation, and ethical compliance, in line with current academic recommendations for observational health research. During the research implementation process, we always adhere to the principle of voluntary participation and fully respect the respondents’ right to make their own choices. All questionnaires are filled out on an anonymous basis to preserve the confidentiality of the study respondents. At the same time, the guidelines expressly indicated that enrolled subjects are entitled to drop out of the investigation at any time during the survey process and will not be affected in any way. The collected data will only be used for this academic research, and all personal information related to nurses will be strictly kept confidential to ensure data security.

### 2.6. Statistical Analysis

After exporting the survey data through the Questionnaire Star platform, it was directly imported into MS Excel software for database construction. After two researchers independently completed data verification, logical validation, and outlier cleaning, *R* statistical software (Version 4.5.2) was employed to conduct the corresponding statistical analyses. *α* = 0.05 was set as the significance threshold for the two‐tailed test, and statistical significance was defined as a *p* value of less than 0.05 [36, 37]. The descriptive statistics section presented the frequency distribution, composition ratio, mean, and standard deviation of each variable. The Pearson correlation analysis method was employed to quantitatively evaluate the correlational relationship among the three core measures. *R* statistical software (Version 4.5.2) was employed to build a structural equation model, which served to analyze the pathway correlations between AI literacy, professional self‐concept, and evidence‐based practice. The mediation effect test adopted the bootstrap technique, which calculated the 95% confidence interval (CI) through 2000 repeated samples. The mediation effect is judged to be statistically significant when the corresponding interval fails to cover the zero point [38, 39]. The evaluation of model fitting degree adopted a comprehensive judgment of multiple indicators: *χ* ^2^/*d* *f*, RMSEA, NFI, GFI, CFI, IFI, and TLI. When the *χ* ^2^/*d* *f* is less than 5, and all other indicators are greater than 0.90, it is judged that the model has a good fitting effect [40, 41].

## 3. Results

The general characteristics of the 471 individuals included in the study sample are displayed in Table 1. Women accounted for 83.65% of the sample, making them the main group. The age group mainly consisted of middle‐aged and young people aged 26–35 years (49.89%), with 21.23% aged ≤ 25 years and only 1.91% aged ≥ 50 years. 59.24% of married individuals and 39.70% of single individuals in terms of marital status. 47.98% of the population had no children, while 22.51% had two or more children. Nearly 70.00% of those had less than 10 years of work experience. Undergraduate education was predominant (69.00%). Pediatrics and other departments each accounted for 25.69% of the department distribution, followed by internal medicine (21.23%) and surgery (18.26%). The professional title structure was relatively balanced, with intermediate professional titles accounting for 32.27%. Clinical nurses accounted for 76.22% of the positions, and 39.70% undertake teaching work. The work shift mainly consists of day and night shifts (62.63%), and in the past 3 years, 40.76% of the research subjects had received three or more training sessions at or above the college level. The overall characteristics were highly educated, middle‐aged, and young people with rich work experience.

**TABLE 1:** General characteristics of study subjects (*n* = 471).

Variables	Categories	N	%
Gender	Female	394	83.65
	Male	77	16.35

Age	≤ 25	100	21.23
	26–35	235	49.89
	36–49	127	26.96
	≥ 50	9	1.91

Years of work experience	Less than 5 years	210	44.59
	6–10 years	110	23.35
	11–15 years	62	13.16
	15–20 years	60	12.74
	More than 20 years	29	6.16

Education background	Associate degree or below	83	17.62
	Bachelor’s degree	325	69.00
	Master’s degree or above	63	13.38

Department	Pediatrics	121	25.69
	Surgery	86	18.26
	Internal medicine	100	21.23
	Obstetrics and gynecology	20	4.25
	Emergency	8	1.70
	NICU/PICU	15	3.18
	Other	121	25.69

Professional title	Nurse	148	31.42
	Nursing specialist	140	29.72
	Senior nursing specialist	152	32.27
	Deputy chief nursing or above	31	6.58

Job position	None	359	76.22
	Nurse manager	67	14.23
	Head nurse or above	45	9.55

Clinical teacher	Yes	187	39.70
	No	284	60.30

Marital status	Single	187	39.70
	Married	279	59.24
	Divorced or widowed	5	1.06

Children number	None	226	47.98
	One	139	29.51
	Two or more	106	22.51

Graveyard shift	day shift	167	35.46
	work shift	295	62.63
	Graveyard shift	9	1.91

Training experience at or above the college level in the past 3 years	0	96	20.38
	1–2	183	38.85
	≥ 3	192	40.76

Table 2 shows the specific scores for nurses’ AI literacy, professional self‐concept, and evidence‐based practice. The average value of AI literacy scores among nurses was 63.34 ± 10.14, with the lowest score for “Ethics.” The average score of nurses’ professional self‐concept was 232.29 ± 42.57, with the lowest score for “Leadership.” The average score for nurses’ evidence‐based practice was 143.05 ± 22.45, with the lowest score for “Knowledge.”

**TABLE 2:** AI literacy, professional self‐concept, and evidence‐based practice scores of nurse participants (*n* = 471).

Variables	Item	Actual score range	Total scores	Mean score
AI literacy	12	12–84	63.34 ± 10.14	5.28 ± 0.85
Awareness	3	3–21	15.70 ± 2.74	5.23 ± 0.91
Usage	3	3–21	15.58 ± 2.94	5.19 ± 0.98
Evaluation	3	3–21	17.29 ± 3.18	5.76 ± 1.06
Ethics	3	3–21	14.77 ± 3.41	4.92 ± 1.14
Professional self‐concept	36	36–288	232.29 ± 42.57	6.45 ± 1.18
Care	6	6–48	38.71 ± 7.31	6.45 ± 1.22
Communications	6	6–48	39.60 ± 7.03	6.60 ± 1.17
Knowledge	5	5–40	33.43 ± 5.68	6.69 ± 1.14
Leadership	6	6–48	36.94 ± 8.59	6.16 ± 1.43
Communication with colleagues	5	5–40	34.09 ± 5.34	6.82 ± 1.07
Self‐esteem	8	8–64	49.52 ± 11.75	6.19 ± 1.47
Evidence‐based practice	35	35–175	143.05 ± 22.45	4.09 ± 0.64
Attitude	10	10–70	42.46 ± 6.34	4.25 ± 0.63
Knowledge	10	10–70	39.10 ± 7.99	3.91 ± 0.80
Skills	9	9–63	37.19 ± 6.04	4.13 ± 0.67
Utilization	6	6–42	24.30 ± 4.39	4.05 ± 0.73

**FIGURE 1:**
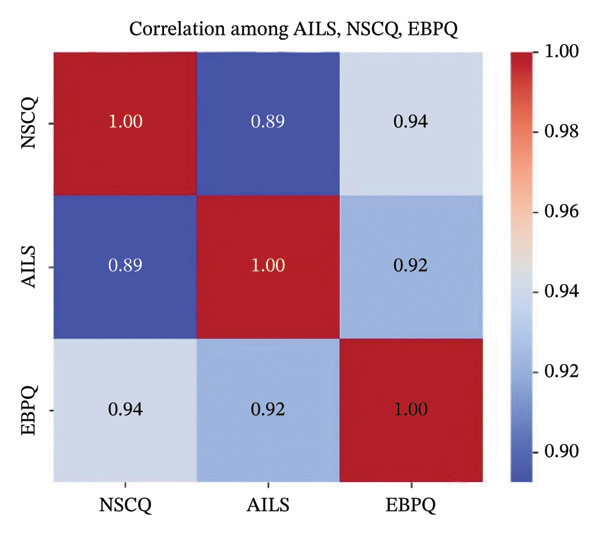
Correlation analysis of nurses’ AI literacy on professional self‐concept and evidence‐based practice.

The outcomes derived from the Pearson correlation analysis are illustrated in Figure 1. The data demonstrated that nurses’ AI literacy was significantly and positively correlated with their professional self‐concept (*r* = 0.89, *p* < 0.001). At the same time, nurses’ professional self‐concept was significantly and positively correlated with their evidence‐based practice (*r* = 0.94, *p* < 0.001). In addition, nurses’ AI literacy was significantly and positively correlated with their evidence‐based practice (*r* = 0.92, *p* < 0.001).

**FIGURE 2:**
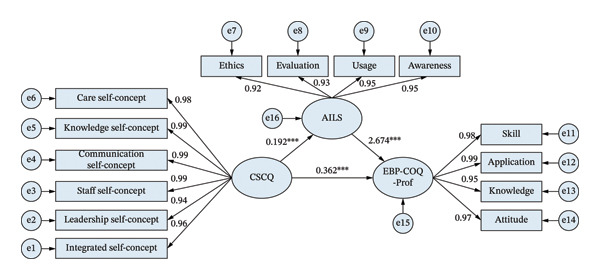
Structural equation model of AI literacy, professional self‐concept, and evidence‐based practice. ∗∗∗*p*  <  0.01.

To explore the mechanism of nurses’ AI literacy in professional self‐concept and evidence‐based practice, a path model with AI literacy as the mediating variable was constructed in this study, as illustrated in Figure 2 and Table 3. Outcomes from the model validation indicated that the mediating model had a good fitting effect on the data: *χ* ^2^/*d* *f* = 2.676 (*p*  <  0.001), RMSEA = 0.021, GFI = 0.938, NFI = 0.913, IFI = 0.928, TLI = 0.953, CFI = 0.979. Figure 2 illustrates the mediation function of AI literacy in linking nurses’ professional self‐concept to evidence‐based practice. All paths in the model had significance (*p*  <  0.001). By way of the intermediary pathway constituted by AI literacy, nurses’ professional self‐concept exerted an indirect effect of 0.226 on evidence‐based practice (95% CI: 0.194–0.260), while the total impact was 0.587 (95% CI: 0.569–0.606). The mediating role of AI literacy contributed 38.5% to the overall effect. In summary, AI literacy played a partial mediating role between nurses’ professional self‐concept and evidence‐based practice: nurses’ professional self‐concept not only directly and positively enhanced their evidence‐based practice but also indirectly promoted the improvement of evidence‐based practice through enhancing AI literacy.

**TABLE 3:** Mediating variable of nurses’ AI literacy on professional self‐concept and evidence‐based practice (*n* = 471).

Mediation model	*β*	SE	LLCI	ULCI	*p*
Total effect	0.587	0.009	0.569	0.606	< 0.001
Direct effect	0.362	0.017	0.328	0.396	< 0.001
Indirect effect	0.226	0.017	0.194	0.260	< 0.001

Abbreviations: LLCI, lower limit confidence interval; SE, standard error; ULCI, upper limit confidence interval.

## 4. Discussion

The results of this study show that the AI literacy level of nurses was outstanding, with a total score of 63.34 ± 10.14. It indicated that nurses not only had good adaptability to digital technology but also exhibited a behavioral tendency to actively use AI tools, providing subject capability support for the implementation of AI in clinical nursing scenarios. This conclusion was consistent with Faiyazuddin’s [42] research viewpoint, which highlighted that the popularity of AI applications among clinical workers had significantly increased, with a positive and empowering effect on optimizing clinical practice processes and improving service quality. In addition, Eachempati et al. [43] further emphasize that integrating AI technology into the healthcare education system, especially in terms of improving information retrieval efficiency, innovating academic research methods, and providing scientific support for clinical decision‐making, could effectively expand the career development path of nursing professionals and offer important opportunities for their ability advancement. However, some studies have yielded contradictory conclusions, suggesting that frontline nurses generally lack systematic AI training and demonstrate low willingness to use intelligent tools due to time constraints and insufficient technical support, resulting in mediocre or even poor AI literacy levels [25, 44]. These inconsistent findings may be attributed to regional differences in medical informatization construction, varying hospital training systems, and disparities in nurses’ exposure to AI applications in daily work.

At the same time, the findings of the research indicated that the level of professional self‐concept among nursing populations was also in a relatively high range, with a total score of 232.29 ± 42.57. Within the scope of nursing, a positive professional self‐concept functions as a core psychological characteristic that brings about multidimensional positive outcomes for the career growth of nursing practitioners: on the one hand, it can strengthen their sense of professional identity and belonging, and improve job satisfaction; in addition, it can effectively alleviate occupational burnout and reduce the willingness to resign [45, 46]. Previous studies have shown that nursing staff possessing a robust sense of professional self‐concept tend to achieve high‐quality service goals in clinical nursing practice and exhibit stronger initiative and adaptability throughout the course of occupational position adjustment and personal growth [47, 48]. Conversely, several cross‐sectional studies have reported relatively low professional self‐concept among nurses, especially those in tertiary hospitals with high workloads and high occupational pressure [15, 49]. This discrepancy may reflect differences in sample composition, such as years of clinical experience, department type, and hospital management support, which moderate the formation and development of professional self‐concept.

With respect to evidence‐based practice, this study measured a total score of 143.05 ± 22.45 for the nursing population, which also showed a high level. Further analysis reveals that nurses’ evidence‐based practice exhibits a robust positive association with core factors, including knowledge accumulation, operational proficiency, attitudes, and practical application level. Positive evidence‐based attitude, systematic knowledge system, proficient operational skills, and high‐frequency practical application together constitute the core elements for improving evidence‐based practice ability [50, 51]. This correlation feature suggests that to continuously improve the evidence‐based practice level of nurses, it is necessary to take the optimization of key variables as the starting point, build a systematic and normalized training system, strengthen nurses’ abilities in cultivating evidence‐based attitudes, updating knowledge, training skills, and guiding practical applications, and lay a solid foundation for the comprehensive implementation of evidence‐based nursing [52, 53]. Nevertheless, conflicting evidence indicates that evidence‐based practice remains at a moderate level in many clinical settings, with barriers including heavy workloads, limited access to literature databases, and inadequate institutional support, which hinder nurses from applying evidence‐based concepts consistently [54]. This divergence implies that evidence‐based practice performance is jointly determined by individual ability and organizational environment, rather than relying solely on personal competency.

Outcomes derived from this study demonstrated that a marked positive correlational relationship existed between nurses’ professional self‐concept and their AI literacy. The conclusion of the present study was highly consistent with existing research findings. As proposed by Falebita and Kok [55], the ongoing utilization of AI‐related applications among nurses would steadily bolster their professional self‐concept with the increasing frequency of hands‐on practice. The improvement of professional self‐concept would drive them to take on challenging work tasks more actively, ultimately forming a virtuous cycle of mutual gain between the two. Mastering AI technology can help nurses efficiently process complex clinical data, achieve interdisciplinary team collaboration, and promote innovative practices in patient care models. The accumulation of such practical opportunities can effectively enhance nurses’ sense of identification and assurance in their professional abilities as well as their potential for career advancement. Therefore, AI literacy can be regarded as a key variable for predicting nurses’ professional self‐concept [56, 57]. In contrast, a few empirical studies have failed to confirm a significant correlation between professional self‐concept and AI literacy, arguing that technical competency and psychological self‐perception belong to relatively independent dimensions and are weakly associated in clinical practice [58, 59]. This inconsistency may stem from differences in measurement tools, sample representativeness, and cultural contexts across studies.

This investigation established that nurses’ evidence‐based practice was significantly and positively correlated with their level of AI literacy. Nurses with high levels of AI literacy tend to be more prone to utilizing AI applications for the systematic collection, in‐depth analysis, and precise interpretation of clinical datasets, thereby significantly improving the accuracy and timelines of nursing decisions and providing key technical support for the clinical implementation of evidence‐based nursing. As noted in the research by Mudallal et al. [55], nurses with high‐level AI literacy demonstrated more remarkable evidence‐based practice competencies, a finding that is highly congruent with the scholarly results of the present study. At the same time, Chong et al. [60] further pointed out that integrating AI technology in the medical field was an effective way to enhance the evidence‐based practice ability of medical staff. Especially in complex nursing scenarios such as intensive care and chronic disease management that require multidimensional data support, the auxiliary value of AI technology was more prominent. In addition, the study by El‐Sayed et al. [61] also confirmed the association between the two, clarifying that AI literacy among nursing staff exerted a marked positive influence on the execution of their evidence‐based practice, thereby supporting the fact that AI application ability had become an indispensable core professional competence for modern nursing staff. However, some scholars have argued that excessive reliance on AI algorithms may reduce nurses’ independent critical thinking and literature evaluation ability, potentially weakening the authenticity and sustainability of evidence‐based practice rather than promoting it [12, 62]. This controversial perspective reminds us that the promotion of AI should be accompanied by standardized training to avoid technical dependence.

In line with recent advances in nursing education research, the mediating role of literacy competencies in technology‐related professional performance has received increasing attention. A recent study demonstrated that digital literacy plays a mediating role, and academic support serves a moderating role, in the relationship between AI usage and creative thinking in nursing students [63]. This contemporary evidence strongly supports the fundamental view that literacy‐related competencies serve as pivotal intermediate variables linking technology engagement to the development of higher‐level professional abilities in nursing. Extending this line of research to clinical nurses, our study further confirms that AI literacy acts as a partial mediator between professional self‐concept and evidence‐based practice. These complementary findings highlight that improving AI literacy and constructing supportive academic and clinical environments are essential to promote the development of innovative and evidence‐based nursing practice in the AI era [11, 64].

The current study verified that nurses’ evidence‐based practice was significantly and positively correlated with their level of professional self‐concept. Durmus et al. [65] pointed out that nursing staff with enhanced professional self‐concept often had more outstanding evidence‐based practice abilities and also demonstrated a stronger sense of responsibility in interpersonal interactions and professional behavior. A parallel conclusion was also reached by Song et al. [66], who noted that the advancement of nurses’ professional self‐concept could exert a notable boosting effect on the development of their evidence‐based practice capabilities. Those nursing professionals with a robust professional self‐concept can not only provide patients with better nursing services but also efficiently transform and apply their nursing expertise. The professional self‐concept of nursing staff is essentially a subjective understanding of their role competence formed during the process of gradually acquiring the specialized knowledge system, skill paradigm, and value concept of their profession [67, 68]. Despite consistent positive findings in the present study, some studies have reported a nonsignificant or even weak association between professional self‐concept and evidence‐based practice, suggesting that the relationship may be moderated by organizational culture, leadership support, and clinical training mechanisms [69, 70]. This indicates that the promotion of evidence‐based practice requires both individual psychological resources and institutional guarantees.

The purpose of this study was to explore the mediating role of AI literacy in the relationship between nurses’ professional self‐concept and evidence‐based practice. In line with this research objective, the current findings verified that AI literacy serves as a partial mediator in the pathway from professional self‐concept to evidence‐based practice, which provides empirical evidence for understanding the internal mechanism among these three key variables in the context of intelligent healthcare.

## 5. Limitation

This research is subject to a number of inherent limitations. First, convenience sampling was adopted, which may reduce sample representativeness and restrict the generalizability of the findings, representing a major methodological limitation of this study. Second, a cross‐sectional design was adopted in the study, which made it difficult to support the inference of causal relationships between variables. Subsequently, longitudinal tracking research can be conducted to further dissect the extended‐duration mechanism of AI literacy on nurses’ professional self‐concept and evidence‐based practice, and accumulate more convincing empirical evidence. Third, the research data is collected through the self‐report method, which can easily lead to social expectation bias, that is, the research subjects tend to present their own state in a positive way that conforms to social value orientation. Future research can introduce objective evaluation tools and observational data collection methods to effectively reduce the interference of such biases. Fourth, the gender composition of the research sample is obviously unbalanced, and the proportion of female nurses is as high as 83.65%, which restricts the promotion of the research conclusion to the male nurse group to a certain extent. A more balanced gender structure survey is needed to make up for this deficiency. Fifth, this study only conducted a comprehensive examination of AI literacy, without subdividing the types of AI tools actually used by nurses. In the future, we can focus on different types of AI‐based tools, including clinical decision platforms, AI‐assisted education tools, social media content recommendation algorithms, etc., and deeply analyze the differentiated impact of various tools on nurses. Finally, the present research was carried out in a particular cultural and organizational context, which may affect nurses’ cognition of AI technology and related learning behaviors. Subsequent research conducted in a multicultural and educational environment will help to enhance the universality of the study outcomes. While the aforementioned limitations should be acknowledged, this research still furnishes useful references for exploring the intrinsic associations between nurses’ AI literacy, professional self‐concept, and evidence‐based practice, which can lay a theoretical and empirical basis for subsequent inquiries in this research area.

## 6. Conclusion

The findings demonstrated that nurses’ AI literacy exhibited a positive association with both their professional self‐concept and evidence‐based practice while also playing a mediation effect in the linkage between professional self‐concept and evidence‐based practice.

This study carries substantial practical implications for nursing education, clinical practice, and nursing management in the era of AI. First, nursing educators should incorporate systematic AI knowledge and skill training into both undergraduate and continuing education curricula to strengthen nurses’ AI literacy and professional self‐concept simultaneously. Second, clinical nursing managers can build supportive and AI‐friendly practice environments to encourage nurses to apply evidence‐based practice with the assistance of AI tools. Third, hospital administrators should develop targeted training programs and standardized guidelines for AI application in clinical nursing, thereby promoting the deep integration of AI technology into routine nursing practice and ultimately improving the quality and efficiency of evidence‐based nursing care.

## Author Contributions

Shanwei Li: methodology, software, formal analysis, conceptualization, and writing–original draft. Liping Wu: validation, formal analysis, supervision, formal analysis, and writing–review and editing. Yan Tang: conceptualization, investigation,data curation, and writing–review and editing.

## Funding

This research received no specific grant from any funding agency in the public, commercial, or not‐for‐profit sectors.

## Ethics Statement

This study was approved by the Ethics Committee of the Children’s Hospital of Chongqing Medical University (approval number: 368/2025). The research adhered to the principles of the Declaration of Helsinki.

## Conflicts of Interest

The authors declare no conflicts of interest.

## Data Availability

The datasets generated during and analyzed during the current study are available from the corresponding author upon reasonable request.
